# Gene Polymorphisms of Parkinson’s Disease Risk Locus and Idiopathic REM Sleep Behavior Disorder

**DOI:** 10.3390/biomedicines13040788

**Published:** 2025-03-24

**Authors:** Min Zhong, Yang Jiao, Aonan Zhao, Mengyue Niu, Jinjun Ran, Jun Liu, Yuanyuan Li

**Affiliations:** 1 Department of Neurology and Institute of Neurology, Ruijin Hospital Affiliated to Shanghai Jiao Tong University School of Medicine, Shanghai 200025, China; neuro_zhongmin@163.com (M.Z.); drjiaoyang@163.com (Y.J.); aonanzhao@gmail.com (A.Z.);; 2 School of Public Health, Shanghai Jiao Tong University School of Medicine, Shanghai 200025, China; jinjunr@sjtu.edu.cn

**Keywords:** idiopathic REM sleep behavior disorder, Parkinson’s Disease, SNP, *SH3GL2*, *COMT*

## Abstract

**Background/Objectives**: Genetic factors play an important role in idiopathic rapid eye movement sleep behavior disorder (iRBD) but have not been fully studied. This study aimed to analyze the Parkinson’s disease (PD)-related genetic loci in iRBD in the southern Chinese population. **Methods**: In this study, we recruited 292 individuals with PD, 62 with iRBD, and 189 healthy controls (HC). Candidate genes were identified primarily from the Parkinson’s Progression Markers Initiative (PPMI) database. Genotypic and allele frequency analyses were conducted to compare the distribution across HC, iRBD, and PD groups. The effects of significant single-nucleotide polymorphisms (SNPs) on gene expression were examined. Clinical manifestations associated with different genotypes were also analyzed. The receiver operating characteristic (ROC) curve and Kaplan–Meier plots were utilized to further verify the diagnostic and predictive value of these SNPs. **Results**: We identified two significant SNPs associated with iRBD: rs13294100 of *SH3GL2* and rs165599 of *COMT*. Clinical scale and polysomnography data analysis indicated that iRBD patients with the GA or AA genotype at the *COMT* rs165599 locus have lower RBDSQ scores and higher sleep efficiency. Moreover, we identified that *COMT* rs165599 and *MCCC1* rs12637471 may play an important role in both PD and iRBD, while *SNCA* rs356181 was different between iRBD and PD. **Conclusions**: Our research revealed that in the southern Chinese demographic, genetic loci in *SH3GL2* and *COMT* were linked to iRBD and may act as potential biomarkers for iRBD risk. Additionally, there is evidence suggesting a partial genetic overlap between iRBD and PD, indicating a shared genetic predisposition.

## 1. Introduction

Idiopathic rapid eye movement sleep behavior disorder (iRBD), characterized by REM sleep without atonia and dream-enactment behavior on polysomnography, is known to be highly associated with α-synucleinopathy neurodegeneration, including Parkinson’s disease (PD), dementia with Lewy bodies, and multiple system atrophy [1]. Up to 91% of iRBD patients eventually convert to a neurodegenerative disorder, mostly a synucleinopathy, over a 14-year follow-up period [2]. Since iRBD can be the prodromal stage of PD, preceding motor symptoms for many years or even decades [3], it is promising that some or a subgroup of iRBD patients may represent an ideal population to predict the conversion of PD [4].

Considering patients with iRBD are more likely to have a family history of dream-enactment behaviors than patients without iRBD [5], genetic factors may play an essential role in iRBD. To date, many gene variants associated with PD, such as *SNCA* and *GBA* (causative genes), as well as *TMEM175*, *INPP5F*, *SCARB2*, *BST1*, and *LAMP3* (risk genes), have also been identified as potential contributors to iRBD in genetic polymorphism analyses [6,7,8,9,10]. Emerging evidence suggests that PD and iRBD may share overlapping genetic components, as certain genes implicated in PD have also been associated with iRBD. Genetic variants have additionally been used as prognostic biomarkers to assess the risk of phenoconversion in iRBD. Although iRBD alone cannot predict the development of a specific α-synucleinopathy subtype [3], investigating the genetic overlap between iRBD and synucleinopathies could provide valuable insights into shared pathogenic mechanisms and early disease markers. Our study focuses on investigating the genetic background of iRBD by analyzing genes already known to be associated with PD. Given that PD is the most common synucleinopathy and more than half of individuals with iRBD eventually develop PD [11], it is important to explore the genetic background shared by iRBD and PD.

Although an increasing number of genes and loci are associated with iRBD [10], these findings cannot be directly applied to the Chinese population due to variations in allele frequencies among various ethnic groups. Additionally, due to the small patient population and complex diagnostic process, genetic analysis of iRBD is far from sufficient.

The objective of our research is to explore the potential overlap of genetic factors between PD and iRBD and to determine whether these factors play a role in diagnosis and prognosis.

## 2. Materials and Methods

### 2.1. Study Design and Workflow

**Figure 1:**
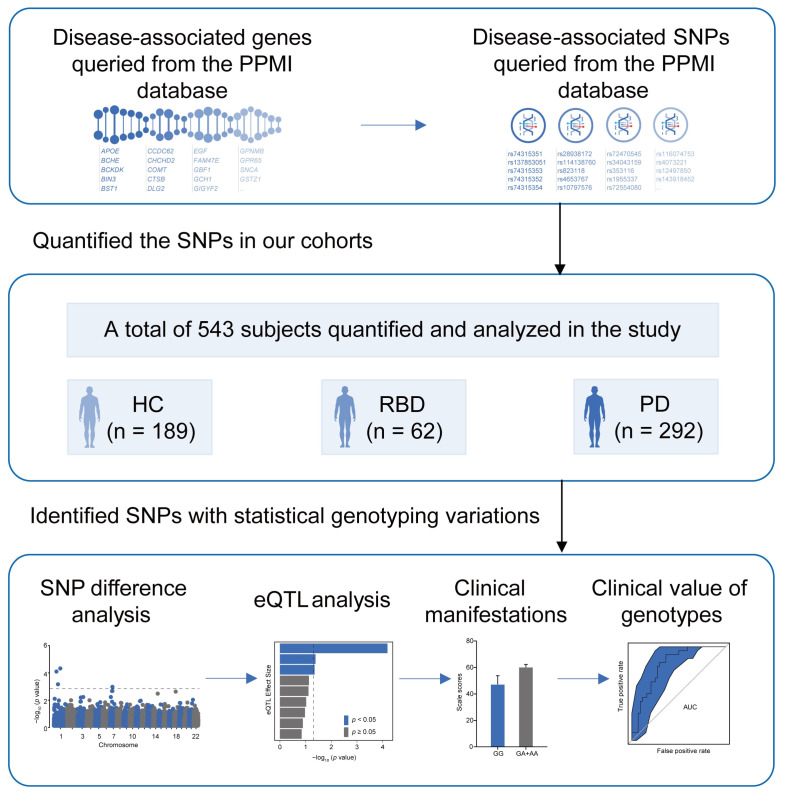
Study design. PPMI; Parkinson’s Progression Markers Initiative; HC; healthy control; iRBD; idiopathic REM Sleep Behavior Disorder; PD; Parkinson’s disease; SNP; single-nucleotide polymorphism; eQTL; expression quantitative trait loci.

To investigate the genetic background of iRBD, we used the identified genes and risk loci in PD primarily queried from the Parkinson’s Progression Markers Initiative (PPMI) database (www.ppmi-info.org/access-data-specimens/data; accessed on 17 September 2024), and applied them to HC, iRBD, and PD participants in the southern Chinese population. We evaluated the potential impact of the susceptible loci on the corresponding gene expression using expression quantitative trait loci (eQTL) analysis. We also studied the clinical manifestations of different genotypes, particularly the alterations in sleep structure, to identify clues that could lead to a better understanding of the pathomechanisms of iRBD and provide a scientific basis for future diagnosis and treatment (Figure 1).

The study provides insights into the genetic basis of iRBD and PD. Genes and SNPs were obtained from the PPMI database and applied to our cohort, which included 189 HC, 62 iRBD, and 292 PD. We analyzed SNP differences, significant eQTL associations, and the clinical impact and utility of genotypes.

### 2.2. Study Participants

Between 2015 and 2022, a total of 543 subjects were recruited from the Department of Neurology at Ruijin Hospital, affiliated with Shanghai Jiao Tong University School of Medicine. This cohort included 292 PD patients, 62 iRBD patients, and 189 healthy controls (HC). The diagnosis of iRBD was based on video polysomnography using the International Classification of Sleep Disorders (ICSD)-III criteria. The exclusion criteria for iRBD included sleep apnea-hypopnea syndrome; the presence of symptoms such as bradykinesia, muscle rigidity, tremor, or postural instability; neurological conditions such as cerebral hemorrhage, cerebral infarction, traumatic brain injury, brain tumors, or central nervous system infections; other sleep disorders or epileptic seizures; and individuals with alcohol intoxication or drug addiction. Approximately 56 of the 62 iRBD patients were followed up from 2015 until they developed dementia, PD, or other movement disorders. PD was diagnosed by movement disorder specialists in accordance with the Movement Disorder Society clinical diagnostic criteria for PD [12]. The exclusion criteria for PD included atypical parkinsonian syndromes, secondary parkinsonism (e.g., drug-induced, stroke), other neurological disorders (e.g., essential tremor, Wilson’s disease), systemic conditions (e.g., thyroid dysfunction), early atypical symptoms (e.g., rapid progression, poor response to levodopa), and imaging abnormalities (e.g., structural lesions). The exclusion criteria for HC typically included neurological disorders, psychiatric conditions, severe systemic diseases, substance abuse, cognitive impairment, recent major surgery or trauma, and other factors, such as pregnancy or severe sensory impairments. This study was approved by the Ethics Committee of Ruijin Hospital affiliated with Shanghai Jiao Tong University School of Medicine (Clinicaltrials.gov, identifier: NCT 04534023) on 1 September 2000. All participants provided written informed consent.

### 2.3. Demographic Information and Clinical Assessment Scales

Demographic information was collected, along with scores from clinical assessment scales at the time of enrollment. For all participants, the REM Sleep Behavior Disorder Screening Questionnaire (RBDSQ) was used to screen for iRBD symptoms. Other questionnaires were administered to evaluate a wide range of symptoms, including the Non-Motor Symptom Questionnaire (NMSQ) for comprehensive non-motor symptoms, the Scale for Outcomes in PD-Autonomic (SCOPA-AUT) for autonomic dysfunction, the Mini-Mental State Examination (MMSE) and the Montreal Cognitive Assessment (MoCA) for cognitive function, the Hamilton Anxiety Scale (HAMA) and Hamilton Depression Scale (HAMD) for anxiety and depression symptoms, and the Sniffin’ Sticks 16-item odor identification test (SS-16) for olfactory function. For all patients, disease duration was collected. The motor subscale of the Movement Disorder Society-sponsored revision of the Unified Parkinson’s Disease Rating Scale (MDS-UPDRS) was used to assess motor function. For PD patients, the levodopa equivalent daily dose (LEDD) was calculated to quantify treatment. The modified Hoehn–Yahr scale was used to assess severity, with mild PD patients defined as those having an H-Y staging below 2.5 [13].

### 2.4. Polysomnography Technique

Polysomnography (PSG) recordings were conducted using a state-of-the-art Compumedics Grael-HD 64 system, equipped with a comprehensive array of sensors. These included six channels for electroencephalogram (EEG) to measure brain activity, two for electrooculogram (EOG) to track eye movements, one for electromyogram (EMG) to record muscle activity, and additional channels for nasal pressure, respiratory effort, oxygen saturation, snoring, electrocardiogram (ECG), and movements of the abdomen, chest, body position, and legs. Infrared cameras were used for continuous video and audio monitoring, providing a detailed record of the patient’s sleep behavior. The EEG leads were meticulously positioned at specific scalp locations—frontal (F3, F4), central (C3, C4), and occipital (O1, O2)—following the internationally recognized 10–20 system. The leads were referenced to mastoid electrodes to ensure accuracy.

The sleep study data were meticulously reviewed by a certified sleep medicine specialist in accordance with the American Academy of Sleep Medicine (AASM) for sleep stage classification and scoring [14]. The PSG analysis provided a comprehensive evaluation of sleep quality, including various objective sleep metrics. These included total sleep time in minutes, duration of wake after sleep onset in minutes, and sleep efficiency as a percentage. Additionally, sleep latency was measured in minutes, and the proportions of Stage N1, N2, and N3 sleep were determined. The analysis also included the calculation of the average heart rate in beats per minute of sleep. Additionally, the apnea-hypopnea index (AHI) was measured to quantify the number of events per hour of sleep, along with the microarousal index, which also counted events per hour of sleep. The periodic limb movement (PLM) index was included to quantify the total number of leg movements throughout the night, providing a complete overview of sleep dynamics.

### 2.5. DNA Extraction, SNPs Selection, and Gene Expression Analysis

Genomic DNA was extracted from isolated peripheral blood mononuclear cells using standard phenol-chloroform extraction procedures. Based on genes and SNPs queried from the PPMI database, we designed a panel to be applied to our participants. All candidate genes were subjected to enrichment analysis using the Kyoto Encyclopedia of Genes and Genomes (KEGG) and Gene Ontology (GO) databases. A custom-designed panel (TWIST Bioscience, South San Francisco, CA, USA) was used to detect the SNPs. Detailed panel information is provided in Table S1. The SNaPshot technique (Applied Biosystems, Foster City, CA, USA) was used for SNP genotyping. eQTL analysis was conducted using the Genotype-Tissue Expression (GTEx) and Brain eQTL Almanac (BrainEAC) datasets. GTEx utilizes whole-genome sequencing and gene expression data, providing a comprehensive transcriptomic and genomic database with a large sample size for analyzing gene expression in the brain and other tissues. In contrast, BrainEAC is brain-specific and contains gene expression array data.

### 2.6. Statistical Analysis

All statistical analyses were conducted using the SPSS 25.0 software package or R 4.3.1. Differences in age between groups were examined with a *t*-test. Chi-square tests were used to compare sex distribution, allele, and genotype frequencies, and to evaluate deviations from Hardy–Weinberg equilibrium (HWE) within the entire cohort. The associations between each SNP and the risk of iRBD (compared to HC), PD (compared to HC), and iRBD compared to PD were estimated using logistic regression analysis after adjusting for age and sex. Five genetic models were applied: codominant, dominant, recessive, overdominant, and additive models. If “A” is defined as the major allele and “a” as a minor allele, the codominant modal is defined as 0 (AA) vs. 1 (Aa) vs. 2 (aa), the dominant model as 1 (aa + Aa) vs. 0 (AA), the recessive model as 1 (aa) vs. 0 (AA + Aa), the overdominant model as 1 (Aa) vs. 0 (aa + AA), and the additive model as 0 (AA) vs. 1 (Aa) vs. 2 (aa). *p* values < 0.05 were considered statistically significant. Multifactorial logistic regression was conducted to analyze gene-gene interactions. Receiver operating characteristic (ROC) curve was generated to analyze the diagnosis effect of the SNPs across different diseases. Kaplan–Meier plots were utilized to assess whether genotype can predict phenotypic conversion.

## 3. Results

### 3.1. Demographic and Clinical Characteristics of Participants

The demographic and clinical characteristics of participants in each group are detailed in Table 1. No significant differences were observed in age or sex distribution among the HC, iRBD, and PD groups. Within the PD group, 73.68% of patients were classified as having mild PD. Significant differences were observed between the three groups on the Non-Motor Rating Scale scores (*p* < 0.001).

### 3.2. PPMI-Derived Genes Enriched in the PD Pathway

We initially conducted enrichment analysis to evaluate the fundamental characteristics of the genes under investigation. Using R software, we performed KEGG and GO functional enrichment analyses on the candidate genes retrieved from the PPMI database. The analysis revealed that a significant majority of the genes were enriched in pathways associated with PD in the KEGG analysis (Figure S1A), highlighting their potential relevance to PD. The genes under investigation were also enriched in numerous pathways in the GO analysis, including positive regulation of cell communication, cell projection, and microtubule binding (Figure S1B–D).

### 3.3. Association of the Two Selected SNPs with iRBD

**Figure 2:**
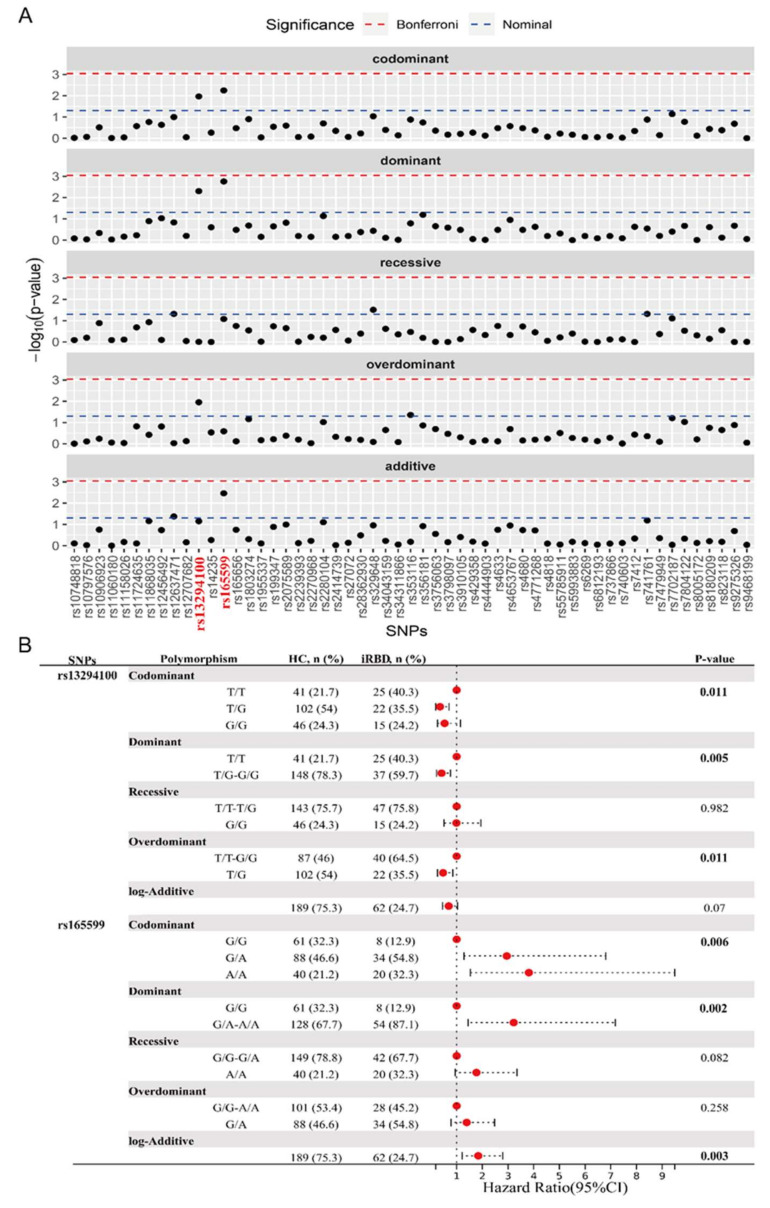
Analysis of genotypic and allele frequency between HC and iRBD groups. (**A**) The genotype and major allele frequencies for all the SNPs. (**B**) Significant statistical differences were observed in the models of rs13294100 of *SH3GL2* and rs165599 of *COMT*. The bold emphasis indicates *p* < 0.05.

To analyze the potential association of these PD-associated genes and SNPs with iRBD, we proceeded to conduct a comparative analysis between HC and iRBD groups. The genotype distribution of all SNPs was consistent with HWE. The genotype and major allele frequencies for all the SNPs are listed in Table S2 and Figure 2A. The overdominant model of rs353116 in *SCN3A*/*SCN2A* (C/T: *p* = 0.044, OR = 1.81) and rs12637471 in *MCCC1* (recessive model: G/G: *p* = 0.048, OR = 2.28; log-additive model: *p* = 0.042, OR = 1.54) was found to be associated with iRBD (Table S2). In addition, as listed in Figure 2B, significant differences were observed in the following models of rs13294100 in *SH3GL2*: the codominant model (T/G: OR = 0.35; G/G: OR = 0.53; *p* = 0.011); the dominant model (T/G-G/G: OR = 0.41, *p* = 0.005); and the overdominant model (T/G: OR = 0.47, *p* = 0.011). Furthermore, significant differences in rs165599 in *COMT* were observed in the following models: the codominant model (G/A: OR = 2.95; A/A: OR = 3.81; *p* = 0.006); the dominant model (G/A-A/A: OR = 3.22, *p* = 0.002); and the log-additive model (OR = 1.84, *p* = 0.003). Thus, two loci (*SH3GL2* rs13294100 and *COMT* rs165599) were significantly associated with iRBD across different genetic models. No significant interaction was observed between *SH3GL2* rs13294100 and *COMT* rs165599 in modulating the risk of iRBD.

### 3.4. The Overlap of SNPs Between Different Groups

**Figure 3:**
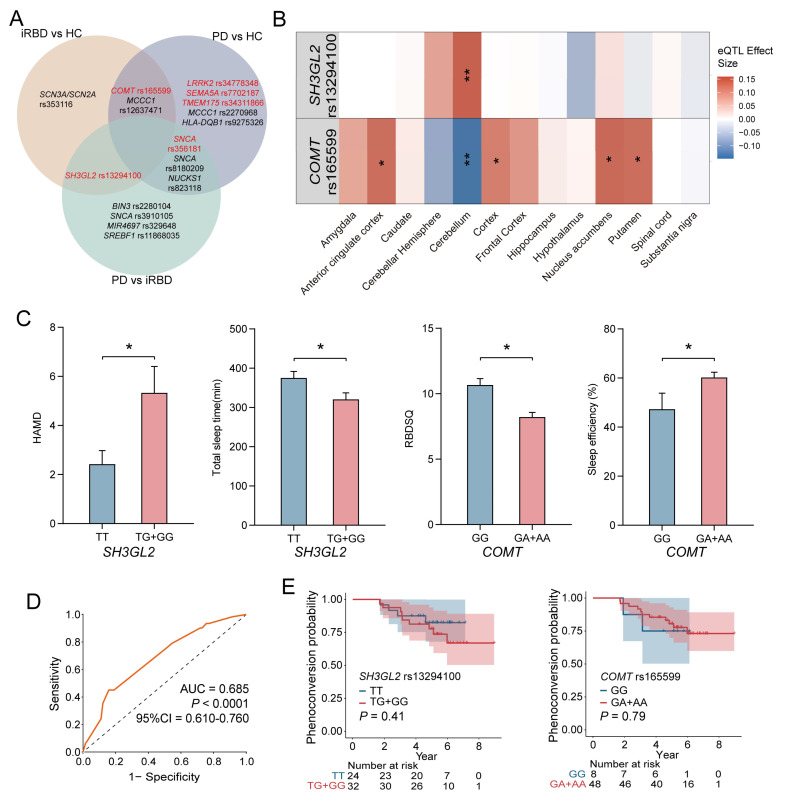
Genetic Associations and Phenotypic Outcomes in iRBD. (**A**) Venn diagram illustrated significant SNPs in the analysis of genotypic and allele frequency between different groups, which are marked red. The genetic risk for PD and iRBD does not overlap completely. *COMT* rs165599 and *MCCC1* rs12637471 may play an important role in both PD and iRBD. *SH3GL2* rs13294100 and *SNCA* rs356181 are significantly different between PD and iRBD. (**B**) In the GTEx database; the rs13294100 correlates most strongly with increased *SH3GL2* expression in the cerebellum (*p* = 1.88 × 10^−4^). The rs165599 correlates with decreased *COMT* expression in the cerebellum (*p* = 9.19 × 10^−4^); and increased *COMT* expression in the putamen (*p* = 0.020); nuclear accumbens (*p* = 0.002); cortex (*p* = 0.031); and anterior cingulate cortex (*p* = 0.028). (**C**) Comparison of clinical assessment and sleep-related phenotypes across different genotypes for *SH3GL2* and *COMT* genes. (**D**) The combination AUC value of all the significant SNPs between HC and iRBD groups was 0.685 (*p* < 0.0001, 95%CI = 0.610–0.760). (**E**) Kaplan–Meier survival curves for the *SH3GL2* rs13294100 and *COMT* rs165599 genotypes. *SH3GL2* rs13294100 and *COMT* rs165599 alone cannot predict iRBD phenoconversion. * represents a *p*-value < 0.05, ** represents a *p*-value < 0.01.

Taking into account differences among ethnic groups, we further investigated the correlation between these SNPs and PD within our cohort, with a particular emphasis on the two loci associated with iRBD. The genotypic and allele frequencies were analyzed between HC and PD groups, as well as iRBD and PD groups (Tables S3 and S4). The genotype and major allele frequencies for all SNPs are listed in Tables S2–S4. Additionally, we found ten PD-associated SNPs, among which three were significant: rs34778348 in *LRRK2*, rs34311866 in *TMEM175*, and rs7702187 in *SEMA5A*. Eight SNPs were significantly different when comparing PD and iRBD, particularly rs356181 in *SNCA*. The combination of these essential SNP could distinguish between other groups, providing evidence that they may serve as potential biomarkers for iRBD or PD diagnosis. As shown in Figure 3A, rs13294100 in *SH3GL2* significantly differed in both two-group comparisons (iRBD vs. HC and iRBD vs. PD), suggesting that it may be a risk factor for iRBD. Another SNP, rs165599 in *COMT*, which was associated with iRBD, was also linked to PD. This suggests that the genetic background in iRBD partially overlaps with that of PD as a whole.

### 3.5. eQTL Insights into Genotype-Dependent Gene Expression

In order to fully understand the functional influence of rs13294100 in *SH3GL2* and rs165599 in *COMT* related to iRBD or PD, eQTL analysis was performed using the GTEx and BrainEAC datasets. As shown in Figure 3B, rs13294100 was most strongly associated with increased *SH3GL2* expression in the cerebellum (*p* = 1.88 × 10^−4^). rs165599 was associated with decreased *COMT* expression in the cerebellum (*p* = 9.19 × 10^−4^) and increased *COMT* expression in the putamen (*p* = 0.020), nucleus accumbens (*p* = 0.002), cortex (*p* = 0.031), and anterior cingulate cortex (*p* = 0.028). The TG genotype of rs13294100 was associated with higher levels of *SH3GL2* in the brain (Figure S4A). The AA genotype of rs165599 was associated with higher levels of *COMT* in the putamen, cortex, and anterior cingulate cortex. The AA genotype of rs165599 was associated with lower levels of *COMT* in the cerebellum. The GA genotype of rs165599 was associated with higher levels of *COMT* in the nucleus accumbens and anterior cingulate cortex (Figure S4B). eQTL analysis revealed that the genotypes rs13294100 and rs165599 significantly modulate *SH3GL2* and *COMT* gene expressions in brain regions.

### 3.6. Genotype-Specific Impacts on iRBD-Related Clinical and Sleep Parameters

We conducted an analysis to identify variations in clinical scale scores and sleep patterns across different genotypes, aiming to elucidate the potential roles of the specific loci *SH3GL2* and *COMT* in iRBD. Using the dominant model, we observed significant differences in both clinical and sleep-related parameters.

For the *SH3GL2* gene, individuals carrying the T/G and G/G genotypes demonstrated significantly poorer scores on the HAMD, indicating a higher prevalence of depressive symptoms, and shorter total sleep durations compared to those with the T/T genotype. Regarding the *COMT* gene, the G/A and A/A genotypes were associated with lower RBDSQ scores and enhanced sleep efficiency, as measured by PSG, suggesting a potential link to more restorative sleep patterns (Figure 3C). Detailed comparative data are provided in Tables S5 and S6. Our findings suggest that the T/G and G/G genotypes of *SH3GL2* were associated with increased depressive symptoms and reduced sleep quality in iRBD, while the G/A and A/A genotypes of *COMT* were linked to better sleep efficiency, indicating a role for these genetic variations in iRBD pathology.

Tables S7 and S8 present genotype-specific effects on clinical indicators and sleep patterns in PD patients. Patients with *COMT* G/A and A/A genotypes exhibited reduced autonomic function, as indicated by lower SCOPA-AUT scores (*p* = 0.02), compared with those with the G/G genotype. No significant differences were observed in other clinical scales or PSG sleep data among genotypes.

### 3.7. ROC Curve and Survival Analysis of SNPs in iRBD Diagnosis and Progression

ROC curve analysis was performed to evaluate the validity of the significant SNPs as potential predictive biomarkers for iRBD diagnosis. The combined AUC value for all significant SNPs between the HC and iRBD groups was 0.685 (*p* < 0.0001, 95%CI = 0.610–0.760), indicating a moderate ability to distinguish between different groups (Figure 3D). The combined AUC value of all the significant SNPs between the HC and PD groups was 0.694 (Figure S5A). The combined AUC value of all the significant SNPs between the RBD and PD groups was 0.743 (Figure S5B).

Among the 62 patients diagnosed with iRBD, data for 56 were available for analysis, which spanned an average follow-up duration of 5.14 years. Within this group, 13 individuals (23%) exhibited neurodegenerative progression, with 12 receiving a PD diagnosis and 1 an MSA diagnosis. To assess the impact of the SNPs under investigation on the transition from iRBD to synucleinopathy, we conducted a Kaplan–Meier survival analysis using dominant genetic models. However, no significant association was found between genotypes and the progression to synucleinopathies, indicating these SNPs are not prognostic for disease course in iRBD (Figure 3E).

## 4. Discussion

From the candidate genes and SNPs identified in the PPMI database, we pinpointed four SNPs linked to iRBD, particularly rs13294100 in *SH3GL2* and rs165599 in *COMT*, both showing significant association. Notably, rs13294100 in *SH3GL2* exhibited substantial differences across both comparative analyses (iRBD vs. HC and iRBD vs. PD), positioning it as a potential iRBD risk factor. Additionally, rs165599 in *COMT*, which has been previously associated with iRBD, was also associated with PD. These findings indicate a shared genetic predisposition between iRBD and PD [15].

Genetic research on iRBD remains limited [15]. A multicenter study found that individuals who carry severe *GBA* variants have an elevated rate of conversion to neurodegeneration, and may experience faster disease progression [16]. An *SNCA* variant was associated with faster conversion to overt synucleinopathies [11]. A recent genome-wide association study (GWAS) identified six iRBD-associated risk loci in five genomic regions: *SCARB2* rs7697073, *INPP5F* rs117896735, *SNCA* rs3756059, *TMEM175* rs34311866, *GBA* rs12752133, and rs76763715 [10]. However, we did not observe an association between these genetic variants and iRBD in our study, which may be caused by ethnic differences and the relatively small sample size. Consistent with the GWAS findings, two endolysosomal system-related genes, *LRRK2* (rs34778348) and *TMEM175* (rs34311866), were found to be associated with PD [10].

One of the iRBD-associated genes identified in our study was *SH3GL2* (SH3 Domain Containing GRB2 Like 2), which encodes endophilin A1. This protein is involved in synaptic vesicle endocytosis and autophagosome by regulating invagination of the nascent vesicles and recruiting synaptojanin 1 [17,18]. *SH3GL2* is a risk gene for PD, and rs13294100 has been associated with PD in a GWAS of the European ancestry population [19,20]. Our study demonstrated that *SH3GL2* (rs13294100) is associated with iRBD and may distinguish iRBD from PD in the Chinese population. Further investigation into the role of endophilin A1 in iRBD is warranted.

*COMT* encodes catechol-O-methyltransferase, which plays an important role in PD treatment. This gene is involved in regulating positive cell communication regulation and cell projection, as indicated by GO functional enrichment analyses. Several *COMT* SNPs have been associated with the risk of PD, including rs4680, rs4633, rs4818, rs6267, rs2075507, and rs362204 [21]. The first two SNPs were validated in Chinese PD patients [22]. rs165599 was initially associated with schizophrenia. In the eQTL analysis, rs165599 was shown to alter the expression of *COMT* in the putamen, nucleus accumbens, cortex, cerebellum, and anterior cingulate cortex in normal tissues. Our findings suggest that *COMT* (rs165599) may play a role in PD and iRBD.

The role of *COMT* in iRBD might diverge from its role in PD. Within the context of PD, the G/A and A/A genotypes of *COMT* are associated with worse autonomic function compared to the G/G genotype, indicating a detrimental effect. In contrast, in iRBD, the G/A and A/A genotypes are linked to more efficient sleep, as indicated by higher RBDSQ scores relative to the G/G genotype, suggesting a protective effect. Variations in *COMT*, particularly the Val158Met (rs4680) polymorphism, have been correlated with various conditions, including schizophrenia and breast cancer [23,24]. These conditions are frequently characterized by immune system dysregulation, suggesting that *COMT* might play a role in their development and progression by modulating the immune response. It is plausible that the role of *COMT* in iRBD might be mediated through immune mechanisms rather than the same enzymatic activity observed in PD.

Studies have shown that *COMT* inhibition may be a promising approach to enhance glioma radiotherapy through a novel immune mechanism. *COMT* inhibition leads to mitochondrial dysfunction in glioma cells, activating the cellular antiviral double-stranded RNA sensing pathway and the type I interferon response to enhance the efficacy of radiotherapy [25]. This suggests that *COMT* may play a role in cancer therapy by influencing immune mechanisms. IL-10 and *COMT* gene polymorphisms were found to interact with cognitive function in patients with schizophrenia, suggesting that *COMT* may influence immune responses through interaction with anti-inflammatory cytokines [26]. Then, more research is needed to see if *COMT* might also be a treatment for iRBD.

Certain constraints within our research should be acknowledged. One of the primary limitations is the modest scale of our sample size. This smaller sample size has resulted in a diminished capacity for SNP detection. As a consequence, the conclusions drawn from our study must be approached with caution. Another significant limitation is the geographical scope of our study. Our research was conducted solely at one location, which may limit its applicability. To confirm the validity of our results, studies across multiple centers are essential. These multicenter studies should also involve larger sample sizes for increased reliability. Additionally, the scope of our SNP analysis was somewhat narrow. We only considered SNPs that were mainly accessible through the PPMI database. This database, while useful, is not comprehensive in its coverage of all relevant SNPs. The limited selection of SNPs from the PPMI database may affect the breadth of our findings. Future research should aim to include a more extensive range of SNPs for a more thorough analysis, which is an aspect we are actively pursuing. While our study successfully identifies genetic associations, it lacks functional validation to confirm the biological mechanisms underlying these SNPs. Incorporating functional assays, such as CRISPR-based gene editing or transcriptomic studies, in future work could elucidate their specific roles in neurodegeneration. By expanding the genetic data considered, we can gain a more accurate understanding of the genetic factors involved.

## 5. Conclusions

In conclusion, the rs13294100 variant of *SH3GL2* and the rs165599 variant of *COMT* were associated with the susceptibility to iRBD in the southern Chinese population and could serve as biomarkers for iRBD. The *COMT* rs165599 polymorphism could be crucially involved in PD and iRBD, with its impact in PD being mediated mainly through enzymatic activity and in iRBD through inflammation. There were some genetic commonalities and differences between PD and iRBD, and larger studies are needed to better elucidate the genetic background of iRBD and PD in different races. Our findings provide valuable insights into the genetic underpinnings of iRBD and its connection to PD, paving the way for future research and clinical advancements.

## Figures and Tables

**Table 1:** Demographic and clinical features of the participants.

	HC	RBD	PD	*p* Value
	(n = 189)	(n = 62)	(n = 292)	
Age (y)	62.77 ± 8.08	63.11 ± 8.72	63.27 ± 8.85	0.806
Sex, n (%)				
Male	112 (59.26%)	39 (62.90%)	156 (53.42%)	0.254
Female	77 (40.74%)	23 (37.10%)	136 (46.58%)	
Disease duration (y)		4.72 ± 4.63	3.95 ± 5.27	0.324
Mild PD, n (%)	-	-	182 (73.68%)	
LEDD	-	-	381.23 ± 271.34	
H-Y stage	-	-	1.75 ± 0.87	
MDS-UPDRS III	-	-	28.17 ± 15.74	
MDS-UPDRS II	-	1.36 ± 2.70	11.43 ± 6.49	**<0.001**
RBDSQ	0.53 ± 0.92	8.52 ± 2.30	4.54 ± 3.48	**<0.001**
MMSE	28.38 ± 1.89	28.32 ± 2.08	27.16 ± 2.97	**<0.001**
MoCA	26.65 ± 3.76	25.96 ± 2.96	22.98 ± 4.43	**<0.001**
HAMA	1.37 ± 1.71	5.95 ± 4.08	7.91 ± 7.32	**<0.001**
HAMD	1.35 ± 1.80	4.13 ± 4.74	5.02 ± 5.32	**<0.001**
SS-16	10.84 ± 2.99	7.70 ± 3.26	7.18 ± 3.18	**<0.001**
SCOPA-AUT	2.05 ± 2.46	7.31 ± 4.99	11.02 ± 7.72	**<0.001**
NMSQ	1.72 ± 1.91	7.28 ± 3.61	8.06 ± 4.24	**<0.001**

HC; healthy control; iRBD; idiopathic REM Sleep Behavior Disorder; PD; Parkinson’s disease; LEDD; levodopa equivalent daily dose; H-Y; Hoehn–Yahr; MDS-UPDRS; Movement Disorder Society-sponsored revision of the Unified Parkinson’s Disease Rating Scale; RBDSQ; REM Sleep Behavior Disorder ScreeFning Questionnaire. MMSE, Mini-Mental State Examination; MoCA, Montreal Cognitive Assessment; HAMA, Hamilton Anxiety Scale; HAMD, Hamilton Depression Scale; SS-16, Sniffin’ Sticks 16-item odor identification test; SCOPA-AUT, Scale for Outcomes in PD-Autonomic; NMSQ, Non-Motor Symptom Questionnaire. Data are presented as mean and SD for continuous variables; and as frequency and % for categorical variables. The bold emphasis in the table indicates *p* < 0.05.

## Data Availability

The data can be available from the corresponding author on reasonable requests.

## Supplementary Materials

The following supporting information can be downloaded at: https://www.mdpi.com/article/10.3390/biomedicines13040788/s1, Figure S1: Clustering analysis of the candidate gene queried from PPMI database; Figure S2: Analysis of genotypic and allele frequency between HCs and PD groups; Figure S3: Analysis of genotypic and allele frequency between PD and iRBD groups; Figure S4: eQTL analysis in BrainEAC database; Figure S5: Diagnosis effect of significant SNPs between different groups; Table S1: Detailed panel information; Table S2: Association of SNPs of candidate genes and odds ratio to iRBD risk; Table S3: Association of SNPs of candidate genes and odds ratio to PD risk; Table S4: Association of SNPs of candidate genes to PD risk among iRBD cases; Table S5: Clinical characteristics of patients with iRBD stratified by *SH3GL2* rs13294100 and *COMT* rs165599 genotypes; Table S6: Sleep parameters in iRBD patients compared across *SH3GL2* rs13294100 and *COMT* rs165599 genotypes; Table S7: Clinical manifestations of different genotypes of PD; Table S8: Sleep structure in different genotypes of PD.

## Abbreviations

The following abbreviations are used in this manuscript:

AASM	American Academy of Sleep Medicine
AHI	apnea-hypopnea index
AUC	area under the curve
BrainEAC	Brain eQTL Almanac
ECG	electrocardiogram
EEG	electroencephalogram
EMG	electromyogram
EOG	electrooculogram
EOPD	early-onset PD
eQTL	expression quantitative trait loci
GO	Gene Ontology
GTEx	Genotype-Tissue Expression
GWAS	genome-wide association study
HAMA	Hamilton Anxiety Scale
HAMD	Hamilton Depression Scale
HC	healthy control
HWE	Hardy–Weinberg equilibrium
H-Y	Hoehn-Yahr
iRBD	Idiopathic rapid eye movement sleep behavior disorder
ISCD	International Classification of Sleep Disorders
KEGG	Kyoto Encyclopedia of Genes and Genomes
LEDD	levodopa equivalent daily dose
MDS-UPDRS	Movement Disorder Society-sponsored revision of the Unified Parkinson’s Disease Rating Scale
MMSE	Mini-Mental State Examination
MoCA	Montreal Cognitive Assessment
NMSQ	Non-Motor Symptom Questionnaire
PD	Parkinson’s disease
PLM	periodic limb movement
PPMI	Parkinson’s Progression Markers Initiative
PSG	polysomnography
RBD	rapid eye movement sleep behavior disorder
RBDSQ	REM Sleep Behavior Disorder Screening Questionnaire
ROC	Receiver operating characteristic
SCOPA-AUT	Scale for Outcomes in PD-Autonomic
SNPs	single-nucleotide polymorphisms
SS-16	Sniffin’ Sticks 16-item odor identification test.
