# Supplementary material for: Gene Polymorphisms of Parkinson’s Disease Risk Locus and Idiopathic REM Sleep Behavior Disorder
Source: Biomedicines. 2025 Mar 24;13(4):788. doi: 10.3390/biomedicines13040788 (PMC12024845; doi:10.3390/biomedicines13040788)
Supplement: Supplementary file 1 [file biomedicines-13-00788-s001.zip › biomedicines-3505266-supplementary.pdf]

Supplementary Results

Analysis of genotypic and allele frequency between HC and PD groups

As listed in **Table S2** and **Figure S2,** and the statistical distinctions were noted in the following models: the rs823118 of *NUCKS1* (recessive model: C/C: OR = 0.6, *P* = 0.037; log-Additive model: OR = 0.73, *P* = 0.019), the rs2270968 of *MCCC1* (dominant model: T/G-G/G: OR = 1.54, *P* = 0.022; overdominant model: T/G: OR = 1.48, *P* = 0.044; log-Additive: OR = 1.54, *P* = 0.042), the rs12637471 of *MCCC1* (dominant model: G/A-G/G: OR = 1.53, *P* = 0.025; log-Additive: OR = 1.40, *P* = 0.018), the rs34311866 of *TMEM175* (codominant model: T/C: OR = 1.47, C/C: OR = 8.07, *P* = 0.011; dominant model: T/C-C/C: OR = 1.63, *P* = 0.023; recessive model: C/C: OR = 7.36, *P* = 0.014; log-Additive: OR = 1.68, *P* = 0.007), the rs356181 of *SNCA* (codominant model: A/G: OR = 0.96, A/A: OR = 0.53, *P* = 0.036; recessive model: A/A: OR = 0.45, *P* = 0.015; log-Additive: OR = 0.7, *P* = 0.013), the rs8180209 of *SNCA* (codominant model: G/A: OR = 0.80, A/A: OR = 0.40, *P* = 0.027; recessive model: A/A: OR = 0.54, *P* = 0.011; log-Additive: OR = 0.75, *P* = 0.035), the rs7702187 of *SEMA5A* (codominant model: T/A: OR = 1.18, T/T: OR = 0.33, *P* = 0.003; recessive model: T/T: OR = 0.31, *P* = 0.001), the rs9275326 of *HLA-DQB1* (codominant model:G/A: OR = 4.2, *P* = 0.00003; dominant model: G/A-A/A: OR = 4.56, *P* = 9.15E-05; log-Additive: OR = 1.42, *P* = 0.018), and the rs34778348 of *LRRK2* (codominant model: T/A: OR = 1.18, T/T: OR = 0.33, *P* = 0.003; overdominant model: G/A: OR = 4.39, *P* = 0.0003).

Analysis of genotypic and allele frequency between iRBD and PD groups

The significant SNPs between patients with iRBD and patients with PD were further analyzed (**Figure S3**), and the significant SNPs were listed in the **Table S3**. The statistical distinctions were noted in the following models: the rs823118 of *NUCKS1* (codominant model: C/T: OR = 1.03, C/C: OR = 0.43, *P* = 0.037; recessive model: C/C: OR = 0.42, *P* = 0.010; log-Additive model: OR = 0.67, *P* = 0.043), the rs356181 of *SNCA* (codominant model: G/A: OR = 0.45, A/A: OR = 0.24, *P* = 0.003; dominant model: G/A-A/A: OR = 0.40, *P* = 0.002; recessive model: A/A: OR = 0.36, *P* = 0.029; log-Additive: OR = 0.48, *P* = 0.0006), the rs8180209 of *SNCA* (log-Additive: OR = 0.66, *P* = 0.044), the rs3910105 of *SNCA* (log-Additive: OR = 0.66, *P* = 0.044), the rs8180209 of *SNCA* (log-Additive: OR = 0.66, *P* = 0.044), the rs13294100 of *SH3GL2* (dominant model: T/G-G/G: OR = 1.95, *P* = 0.024;overdominant model: T/G: OR = 1.82, *P* = 0.036), the rs329648 of *MIR4697* (recessive model: T/T: OR = 4.09, *P* = 0.021; log-Additive: OR = 1.51, *P* = 0.050), and the rs329648 of *SREBF1* (recessive model: G/G: OR = 0.20, *P* = 0.033).

Supplementary Figures and Tables

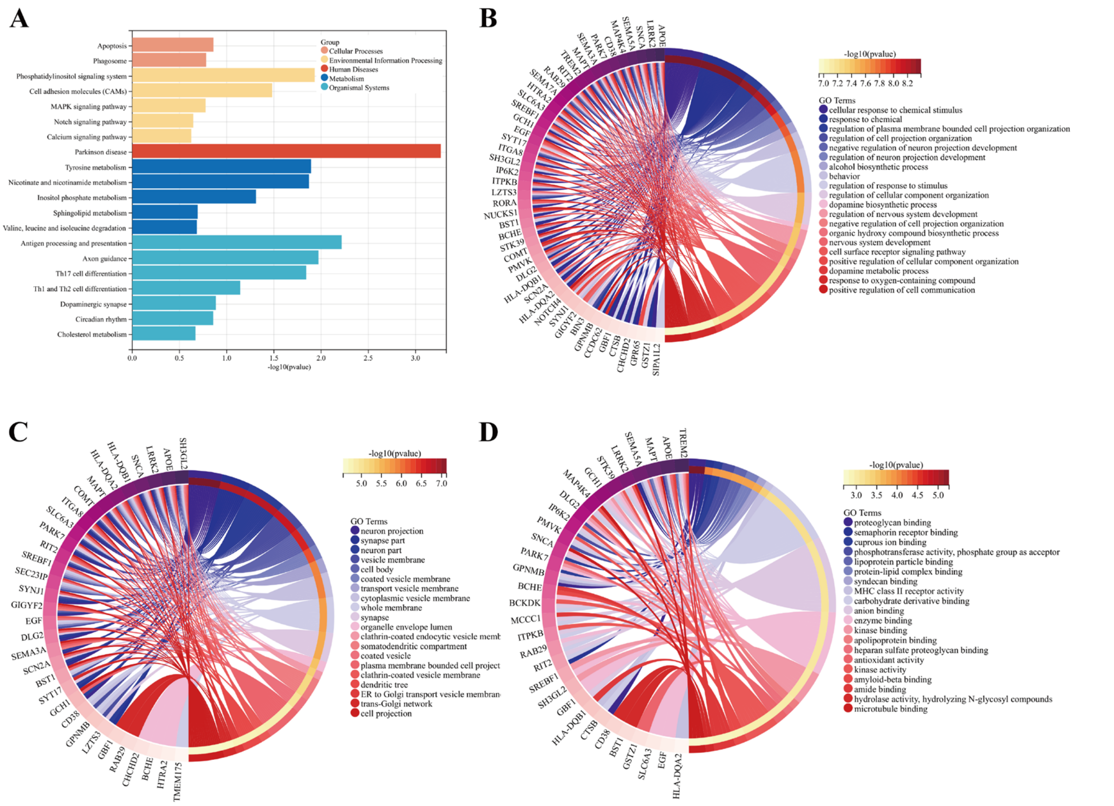

Figure S1. Clustering analysis of the candidate gene queried from PPMI database. (A) Top 20 of enriched KEGG pathways. **(B)** Top 20 of enriched cell component GO functional enrichment analyses. **(C)** Top 20 of enriched molecular function GO functional enrichment analyses. **(D)** Top 20 of enriched biological process GO functional enrichment analyses.

A

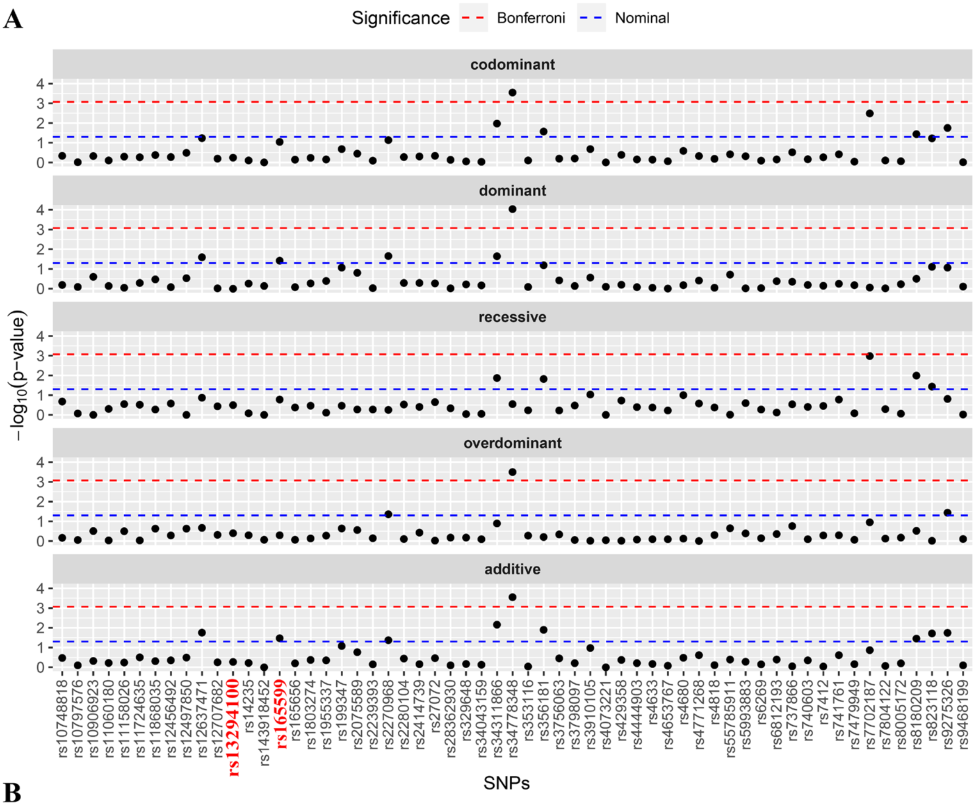

B

SNPs	Polymorphism		HC, n (%)	PD, n (%)	P-value
rs13294100	**Codominant**				
		G/G	46 (24.3)	71 (24.3)	0.572
		T/G	102 (50.0)	146 (50.0)	
		T/T	41 (21.7)	75 (25.7)	
	**Dominant**				
		G/G	46 (24.3)	71 (24.3)	0.995
		T/G-G/G	143 (75.7)	221 (75.7)	
	**Recessive**				
		G/G-T/G	148 (78.3)	217 (74.3)	0.315
		T/T	41 (21.7	75 (25.7)	
	**Overdominan**				
		G/G-T/T	87 (46)	146 (50.0)	0.395
		T/G	102 (54)	146 (50.0)	
	**log-Additive**				
			189 (39.3)	292 (60.7)	0.537
rs165599	**Codominant**				
		G/G	61 (32.3)	69 (23.6)	0.09
		G/A	88 (46.6)	145 (49.7)	
		A/A	40 (21.2)	78 (26.7)	
	**Dominant**				
		G/G	61 (32.3)	69 (23.6)	**0.038**
		G/A-A/A	128 (67.7)	223 (76.4)	
	**Recessive**				
		G/G-G/A	149 (78.8)	214 (73.3)	0.165
		A/A	40 (21.2)	78 (26.7)	
	**Overdominan**				
		G/G-A/A	101 (53.4)	28 (45.2)	0.258
		G/A	88 (46.6)	34 (54.8)	
	**log-Additive**				
			189 (75.3)	62 (24.7)	**0.003**

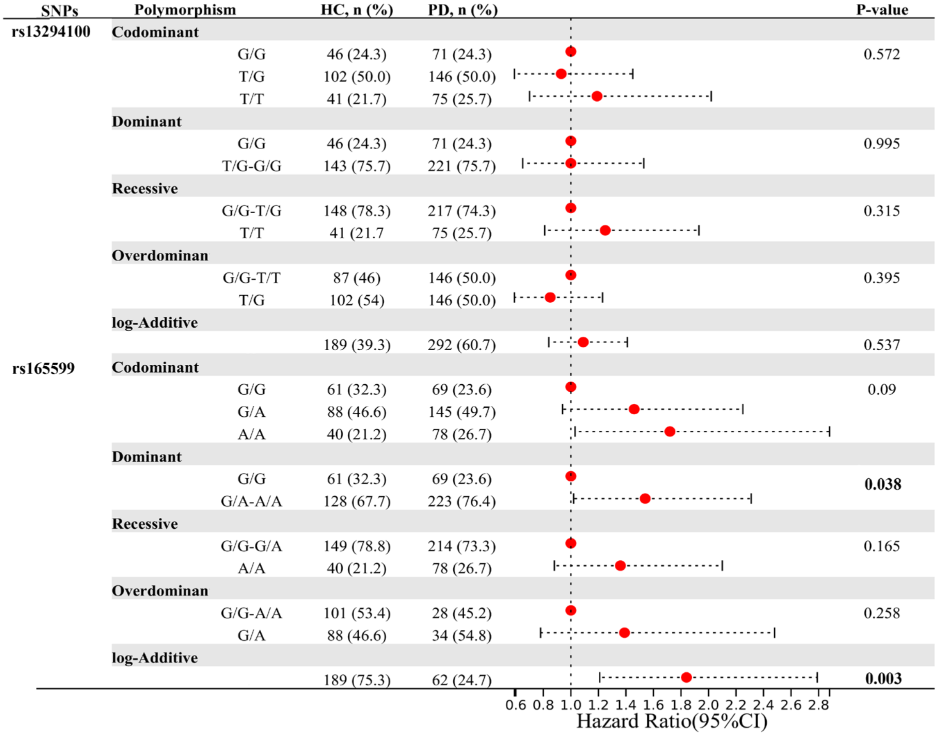

Figure S2. Analysis of genotypic and allele frequency between HCs and PD groups. (A) The genotype and major allele frequencies for all the SNPs. rs165599 of *COMT* and rs13294100 of *SH3GL2* were marked bold red text. **(B)** Significant statistical differences were observed in the rs165599 of *COMT* (in bold red text), but no significant statistical differences were observed in the rs13294100 of *SH3GL2* (in bold red text).

A

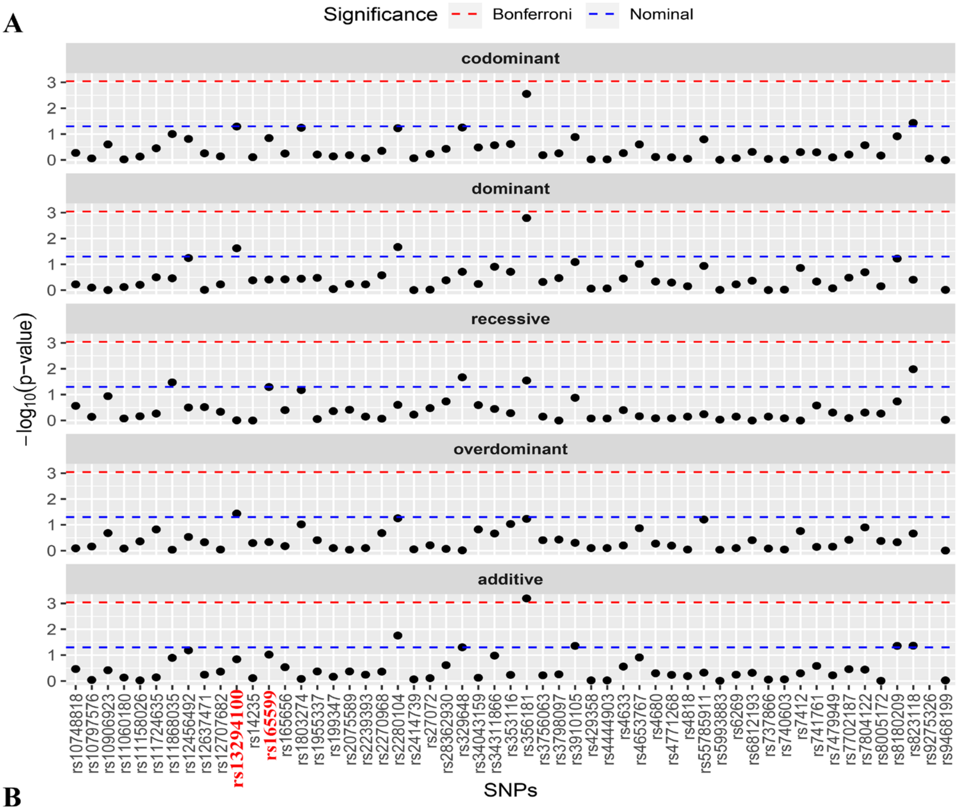

B

SNPs	Polymorphism		RBD, n (%)	PD, n (%)	P-value
rs13294100	Codominant				
		T/T	25 (40.3)	71 (24.3)	0.051
		T/G	22 (35.5)	146 (50.0)	
		G/G	15 (24.2)	75 (25.7)	
	Dominant				
		T/T	25 (40.3)	75 (25.7)	**0.024**
		T/G-G/G	37 (59.7)	217 (74.3)	
	Recessive				
		T/T-G/G	47 (75.8)	221 (75.7)	0.984
		T/G	15 (24.2)	71 (24.3)	
	Overdominant				
		G/G-T/T	40 (64.5)	146 (50.0)	**0.036**
		T/G	22 (35.5)	146 (50.0)	
	log-Additive				
			62 (17.5)	292 (82.5)	0.144
rs165599	Codominant				
		A/A	20 (32.3)	78 (26.7)	0.143
		G/A	34 (54.8)	145 (49.7)	
		G/G	8 (12.9)	69 (23.6)	
	Dominant				
		A/A	20 (32.3)	78 (26.7)	0.381
		G/A-G/G	42 (87.1)	214 (73.3)	
	Recessive				
		A/A-G/A	54(87.1)	223 (76.4)	0.051
		G/G	8 (12.9)	69 (23.6)	
	Overdominant				
		A/A-G/G	28 (45.2)	147 (50.3)	0.458
		G/A	34 (54.8)	145 (49.7)	
	log-Additive				
			62 (17.5)	292 (82.5)	0.095

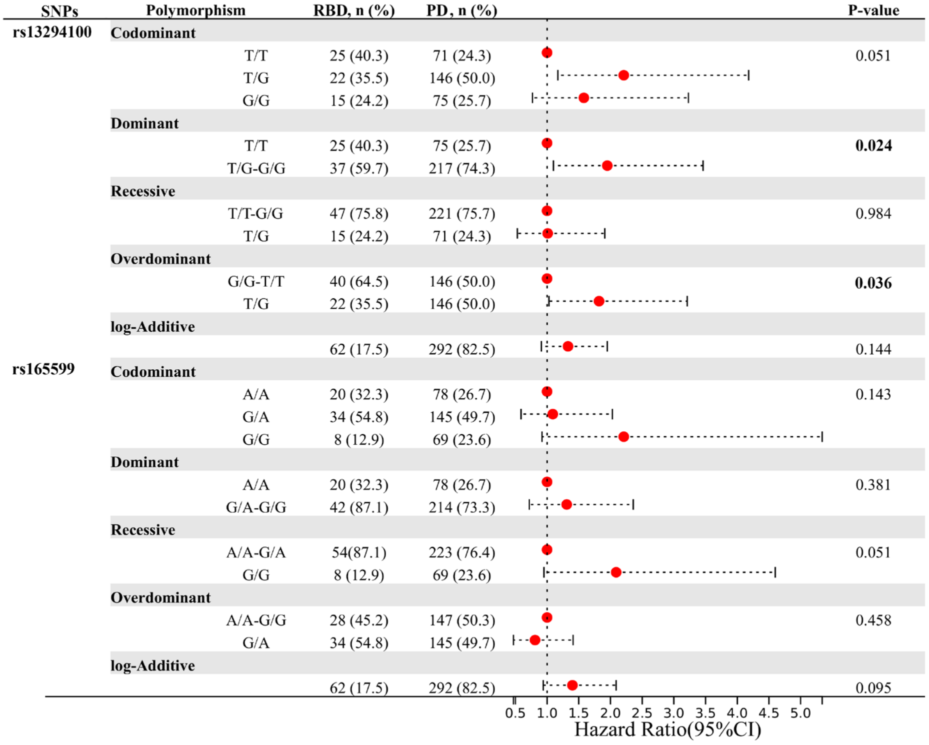

Figure S3. Analysis of genotypic and allele frequency between PD and iRBD groups. (A) The genotype and major allele frequencies for all the SNPs. rs165599 of *COMT* and rs13294100 of *SH3GL2* were marked bold red text. **(B)** Significant statistical differences were observed in the rs13294100 of *SH3GL2*, but no significant statistical differences were observed in the rs165599 of *COMT*.

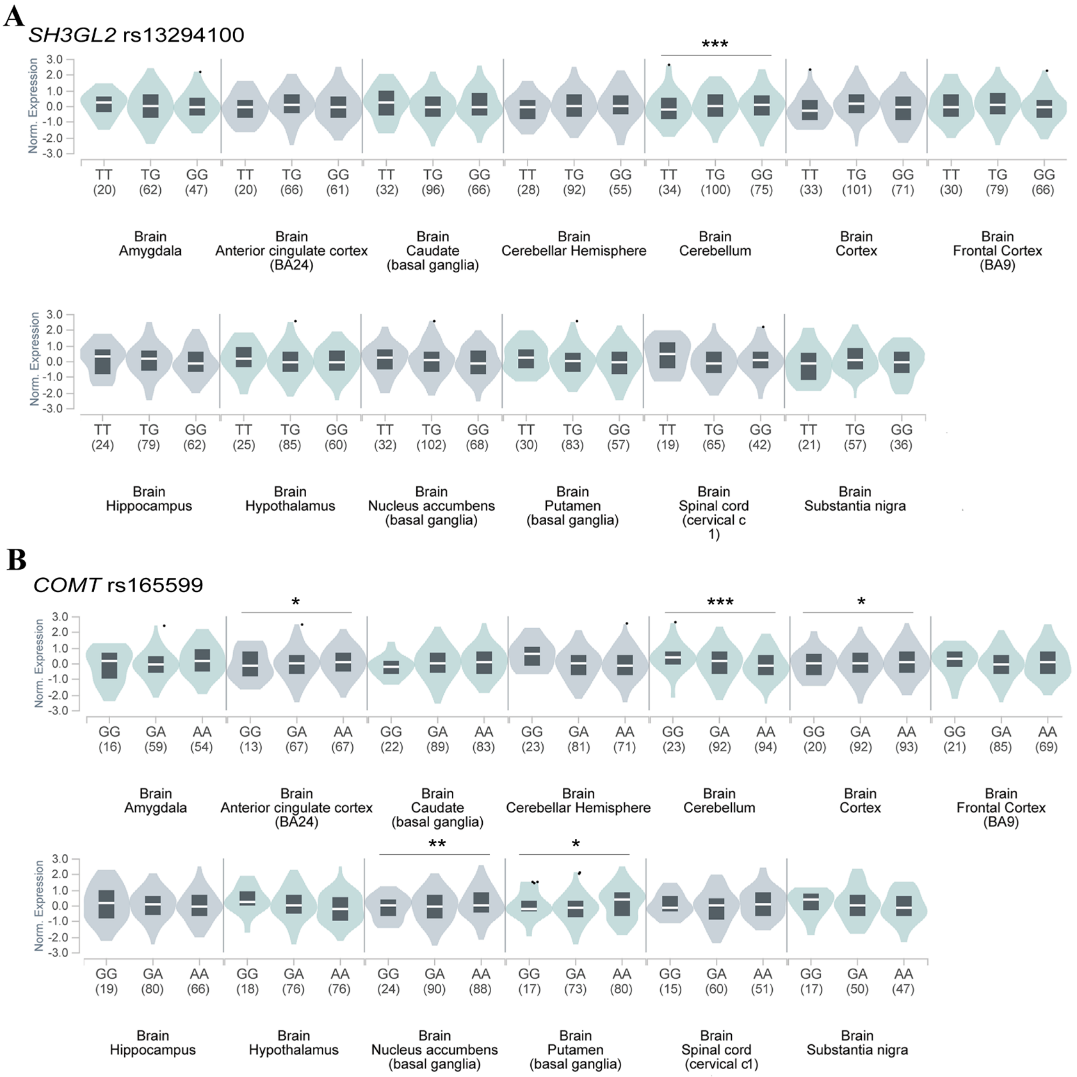

Figure S4. eQTL analysis in BrainEAC database. The TG genotype of 13294100 was associated with the higher levels of *SH3GL2* in the brain. D. In BrainEAC database; the AA genotype of rs165599 was associated with the higher levels of *COMT* in the putamen; cortex; and the anterior cingulate cortex; The AA genotype of rs165599 was associated with the lower levels of *COMT* in the cerebellum; the GA genotype of rs165599 was associated with the higher levels of *COMT* in the nuclear accumbens and the anterior cingulate cortex. *$p < 0.05$; **$p < 0.01$; ***$p < 0.001$.

Table S1. Detailed panel information (Provided in excel sheet format).

Table S2. Association of SNPs of candidate genes and odds ratio to iRBD risk.

Gene SNP	Polymorphism	HC, *n* (%)	iRBD, *n* (%)	OR (95%CI)	*p*-value
SCN3A/SCN2A	**Codominant**				
**rs353116**	T/T	70 (37)	17 (27.4)	1	0.1314
	C/T	85 (45)	37 (59.7)	1.79 (0.93 – 3.45)	
	C/C	34 (18)	8 (12.9)	0.97 (0.38 – 2.47)	
	**Dominant**				
	T/T	70 (37)	17 (27.4)	1	0.1618
	C/T-C/C	119 (63)	45 (72.6)	1.56 (0.83 – 2.93)	
	**Recessive**				
	T/T-C/T	155 (82)	54 (87.1)	1	0.3406
	C/C	34 (18)	8 (12.9)	0.68 (0.29 – 1.55)	
	**Overdominant**				
	T/T-C/C	104 (55)	25 (40.3)	1	**0.04403**
	C/T	85 (45)	37 (59.7)	1.81 (1.01 – 3.24)	
	**log-Additive**				
		189 (75.3)	62 (24.7)	1.1 (0.73 – 1.66)	0.6559
MCCC1	**Codominant**				
**rs12637471**	A/A	90 (47.6)	23 (37.1)	1	0.1005
	G/A	81 (42.9)	27 (43.5)	1.3 (0.69 – 2.45)	
	G/G	18 (9.5)	12 (19.4)	2.61 (1.10 – 6.18)	
	**Dominant**				
	A/A	90 (47.6)	23 (37.1)	1	0.1463
	G/A-G/G	99 (52.4)	39 (62.9)	1.54 (0.86 – 2.78)	
	**Recessive**				
	A/A-G/A	171 (90.5)	50 (80.6)	1	**0.04786**
	G/G	18 (9.5)	12 (19.4)	2.28 (1.03 – 5.05)	
	**Overdominant**				
	A/A-G/G	108 (57.1)	35 (56.5)	1	0.924
	G/A	81 (42.9)	27 (43.5)	1.03 (0.58 – 1.83)	
	**log-Additive**				
		189 (75.3)	62 (24.7)	1.54 (1.02 – 2.34)	**0.04219**
SH3GL2	**Codominant**				
**rs13294100**	T/T	41 (21.7)	25 (40.3)	1	**0.0107**
	T/G	102 (54)	22 (35.5)	0.35 (0.18 – 0.70)	
	G/G	46 (24.3)	15 (24.2)	0.53 (0.25 – 1.15)	
	**Dominant**				
	T/T	41 (21.7)	25 (40.3)	1	**0.004928**
	T/G-G/G	148 (78.3)	37 (59.7)	0.41 (0.22 – 0.76)	
	**Recessive**				
	T/T-T/G	143 (75.7)	47 (75.8)	1	0.9816
	G/G	46 (24.3)	15 (24.2)	0.99 (0.51 – 1.94)	
	**Overdominant**				
	T/T-G/G	87 (46)	40 (64.5)	1	**0.01105**
	T/G	102 (54)	22 (35.5)	0.47 (0.26 – 0.85)	
	**log-Additive**				
		189 (75.3)	62 (24.7)	0.69 (0.45 – 1.04)	0.07021
COMT	**Codominant**				
**rs165599**	G/G	61 (32.3)	8 (12.9)	1	**0.005616**
	G/A	88 (46.6)	34 (54.8)	2.95 (1.28 – 6.80)	
	A/A	40 (21.2)	20 (32.3)	3.81 (1.53 – 9.49)	
	**Dominant**				
	G/G	61 (32.3)	8 (12.9)	1	**0.00175**
	G/A-A/A	128 (67.7)	54 (87.1)	3.22 (1.44 – 7.18)	
	**Recessive**				
	G/G-G/A	149 (78.8)	42 (67.7)	1	0.08212
	A/A	40 (21.2)	20 (32.3)	1.77 (0.94 – 3.35)	
	**Overdominant**				
	G/G-A/A	101 (53.4)	28 (45.2)	1	0.2577
	G/A	88 (46.6)	34 (54.8)	1.39 (0.78 – 2.48)	
	**log-Additive**				
		189 (75.3)	62 (24.7)	1.84 (1.21 – 2.79)	**0.003455**

Table S3. Association of SNPs of candidate genes and odds ratio to PD risk.

Gene SNP	Polymorphism	HC, *n* (%)	PD, *n* (%)	OR (95%CI)	*p*-value
NUCKS1	**Codominant**				
**rs823118**	T/T	54 (28.6)	106 (36.3)	1	0.05876
	C/T	93 (49.2)	143 (49)	0.78 (0.52 − 1.19)	
	C/C	42 (22.2)	43 (14.7)	0.52 (0.30 − 0.89)	
	**Dominant**				
	T/T	54 (28.6)	106 (36.3)	1	0.07733
	C/T-C/C	135 (71.4)	186 (63.7)	0.7 (0.47 − 1.04)	
	**Recessive**				
	T/T-C/T	147 (77.8)	249 (85.3)	1	**0.0369**
	C/C	42 (22.2)	43 (14.7)	0.6 (0.38 − 0.97)	
	**Overdominant**				
	T/T-C/C	96 (50.8)	149 (51)	1	0.9601
	C/T	93 (49.2)	143 (49)	0.99 (0.69 − 1.43)	
	**log-Additive**				
	0,1,2	189 (39.3)	292 (60.7)	0.73 (0.56 − 0.95)	**0.01915**
MCCC1	**Codominant**				
**rs2270968**	T/T	112 (59.3)	142 (48.6)	1	0.07255
	T/G	63 (33.3)	124 (42.5)	1.55 (1.05 − 2.30)	
	G/G	14 (7.4)	26 (8.9)	1.46 (0.73 − 2.94)	
	**Dominant**				
	T/T	112 (59.3)	142 (48.6)	1	**0.0223**
	T/G-G/G	77 (40.7)	150 (51.4)	1.54 (1.06 − 2.22)	
	**Recessive**				
	T/T-T/G	175 (92.6)	266 (91.1)	1	0.5589
	G/G	14 (7.4)	26 (8.9)	1.22 (0.62 − 2.40)	
	**Overdominant**				
	T/T-G/G	126 (66.7)	168 (57.5)	1	**0.04391**
	T/G	63 (33.3)	124 (42.5)	1.48 (1.01 − 2.16)	
	**log-Additive**				
	0,1,2	189 (39.3)	292 (60.7)	1.35 (1.01 − 1.81)	**0.04192**
MCCC1	**Codominant**				
**rs12637471**	A/A	90 (47.6)	109 (37.3)	1	0.05791
	G/A	81 (42.9)	142 (48.6)	1.45 (0.98 − 2.14)	
	G/G	18 (9.5)	41 (14)	1.88 (1.01 − 3.50)	
	**Dominant**				
	A/A	90 (47.6)	109 (37.3)	1	**0.02543**
	G/A-G/G	99 (52.4)	183 (62.7)	1.53 (1.05 − 2.21)	
	**Recessive**				
	A/A-G/A	171 (90.5)	251 (86)	1	0.1346
	G/G	18 (9.5)	41 (14)	1.55 (0.86 − 2.79)	
	**Overdominant**				
	A/A-G/G	108 (57.1)	150 (51.4)	1	0.2145
	G/A	81 (42.9)	142 (48.6)	1.26 (0.87 − 1.82)	
	**log-Additive**				
	0,1,2	189 (39.3)	292 (60.7)	1.4 (1.06 − 1.85)	**0.01763**
TMEM175	**Codominant**				
**rs34311866**	T/T	149 (78.8)	203 (69.5)	1	**0.01061**
	T/C	39 (20.6)	78 (26.7)	1.47 (0.95 − 2.28)	
	C/C	1 (0.5)	11 (3.8)	8.07 (1.03 − 63.17)	
	**Dominant**				
	T/T	149 (78.8)	203 (69.5)	1	**0.02289**
	T/C-C/C	40 (21.2)	89 (30.5)	1.63 (1.06 − 2.51)	
	**Recessive**				
	T/T-T/C	188 (99.5)	281 (96.2)	1	**0.01361**
	C/C	1 (0.5)	11 (3.8)	7.36 (0.94 − 57.43)	
	**Overdominant**				
	T/T-C/C	150 (79.4)	214 (73.3)	1	0.1264
	T/C	39 (20.6)	78 (26.7)	1.4 (0.91 − 2.17)	

	**log-Additive**				
	0,1,2	189 (39.3)	292 (60.7)	1.68 (1.14 − 2.48)	**0.006884**
SNCA	**Codominant**				
**rs356181**	G/G	86 (45.5)	158 (54.1)	1	**0.02689**
	G/A	80 (42.3)	117 (40.1)	0.8 (0.54 − 1.17)	
	A/A	23 (12.2)	17 (5.8)	0.4 (0.20 − 0.79)	
	**Dominant**				
	G/G	86 (45.5)	158 (54.1)	1	0.06503
	G/A-A/A	103 (54.5)	134 (45.9)	0.71 (0.49 − 1.02)	
	**Recessive**				
	G/G-G/A	166 (87.8)	275 (94.2)	1	**0.01516**
	A/A	23 (12.2)	17 (5.8)	0.45 (0.23 − 0.86)	
	**Overdominant**				
	G/G-A/A	109 (57.7)	175 (59.9)	1	0.6228
	G/A	80 (42.3)	117 (40.1)	0.91 (0.63 − 1.32)	
	**log-Additive**				
	0,1,2	189 (39.3)	292 (60.7)	0.7 (0.52 − 0.93)	**0.01266**
SNCA	**Codominant**				
**rs8180209**	G/G	57 (30.2)	101 (34.6)	1	**0.03627**
	A/G	88 (46.6)	150 (51.4)	0.96 (0.63 − 1.46)	
	A/A	44 (23.3)	41 (14)	0.53 (0.31 − 0.90)	
	**Dominant**				
	G/G	57 (30.2)	101 (34.6)	1	0.3109
	A/G-A/A	132 (69.8)	191 (65.4)	0.82 (0.55 − 1.21)	
	**Recessive**				
	G/G-A/G	145 (76.7)	251 (86)	1	**0.01019**
	A/A	44 (23.3)	41 (14)	0.54 (0.34 − 0.86)	
	**Overdominant**				
	G/G-A/A	101 (53.4)	142 (48.6)	1	0.3027
	A/G	88 (46.6)	150 (51.4)	1.21 (0.84 − 1.75)	
	**log-Additive**				
		189 (39.3)	292 (60.7)	0.75 (0.58 − 0.98)	**0.03494**
SEMA5A	**Codominant**				
**rs7702187**	A/A	97 (51.3)	152 （52.1）	1	**0.003211**
	T/A	69 (36.5)	128 （43.8）	1.18 (0.80 − 1.75)	
	T/T	23 (12.2)	12 （4.1）	0.33 (0.16 − 0.70)	
	**Dominant**				
	A/A	97 (51.3)	152 （52.1）	1	0.8753
	T/A-T/T	92 (48.7)	140 （47.9）	0.97 (0.67 − 1.40)	
	**Recessive**				
	A/A-T/A	166 (87.8)	280 （95.9）	1	**0.001041**
	T/T	23 (12.2)	12 （4.1）	0.31 (0.15 − 0.64)	
	**Overdominant**				
	A/A-T/T	120 (63.5)	164 （56.2）	1	0.1095
	T/A	69 (36.5)	128 （43.8）	1.36 (0.93 − 1.98)	
	**log-Additive**				
		189 (39.3)	292 （60.7）	0.8 (0.60 − 1.07)	0.1337
HLA-DQB1	**Codominant**				
**rs9275326**	C/C	170 (89.9)	247 （84.6）	1	**0.01766**
	C/T	17 (9)	45 （15.4）	1.82 (1.01 − 3.29)	
	T/T	2 (1.1)	0 （0）	0 (0.00 − Inf)	
	**Dominant**				
	C/C	170 (89.9)	247 （84.6）	1	0.08607
	C/T-T/T	19 (10.1)	45 （15.4）	1.63 (0.92 − 2.88)	
	**Recessive**				
	C/C-C/T	187 (98.9)	292 （100）	1	0.1539
	T/T	2 (1.1)	0 （0）	0 (0.00 − Inf)	
	**Overdominant**				
	C/C-T/T	172 (91)	247 （84.6）	1	**0.03628**
	C/T	17 (9)	45 （15.4）	1.84 (1.02 − 3.33)	
	**log-Additive**				
		189 (39.3)	292 （60.7）	1.42 (0.83 − 2.44)	**0.01766**
LRRK2	**Codominant**				
**rs34778348**	G/G	183 (96.8)	254 （87）	1	**0.0002793**

	G/A	6 (3.2)	35 （12）	4.2 (1.73 – 10.20)	
	A/A	0 (0)	3 （1）	0 (0.00 – Inf)	
	**Dominant**				
	G/G	183 (96.8)	254 （87）	1	**9.15E-05**
	G/A-A/A	6 (3.2)	38 （13）	4.56 (1.89 – 11.02)	
	**Recessive**				
	G/G-G/A	189 (100)	289 （99）	1	0.2829
	A/A	0 (0)	3 （1）	0 (0.00 – Inf)	
	**Overdominant**				
	G/G-A/A	183 (96.8)	257 （88）	1	**0.0003152**
	G/A	6 (3.2)	35 （12）	4.15 (1.71 – 10.08)	
	**log-Additive**				
		189 (39.3)	292 （60.7）	4.39 (1.85 – 10.42)	**0.0002793**
COMT	**Codominant**				
**rs165599**	G/G	61 (32.3)	69 （23.6）	1	0.09028
	G/A	88 (46.6)	145 （49.7）	1.46 (0.94 – 2.25)	
	A/A	40 (21.2)	78 （26.7）	1.72 (1.03 – 2.88)	
	**Dominant**				
	G/G	61 (32.3)	69 （23.6）	1	**0.0381**
	G/A-A/A	128 (67.7)	223 （76.4）	1.54 (1.02 – 2.31)	
	**Recessive**				
	G/G-G/A	149 (78.8)	214 （73.3）	1	0.1645
	A/A	40 (21.2)	78 （26.7）	1.36 (0.88 – 2.10)	
	**Overdominant**				
	G/G-A/A	101 (53.4)	147 （50.3）	1	0.5068
	G/A	88 (46.6)	145 （49.7）	1.13 (0.78 – 1.63)	
	**log-Additive**				
		189 (39.3)	292 （60.7）	1.32 (1.02 – 1.71)	**0.03379**

Table S4. Association of SNPs of candidate genes to PD risk among iRBD cases.

Gene<br>SNP	Polymorphism	iRBD, *n* (%)	PD, *n* (%)	OR<br>(95%CI)	*p*-value
NUCKS1	**Codominant**				
**rs823118**	T/T	19 (30.6)	106 (36.3)	1	**0.0371**
	C/T	25 (40.3)	143 (49)	1.03 (0.54 – 1.96)	
	C/C	18 (29)	43 (14.7)	0.43 (0.21 – 0.89)	
	**Dominant**				
	T/T	19 (30.6)	106 (36.3)	1	0.3932
	C/T-C/C	43 (69.4)	186 (63.7)	0.78 (0.43 – 1.40)	
	**Recessive**				
	T/T-C/T	44 (71)	249 (85.3)	1	**0.0103**
	C/C	18 (29)	43 (14.7)	0.42 (0.22 – 0.80)	
	**Overdominant**				
	T/T-C/C	37 (59.7)	149 (51)	1	0.2139
	C/T	25 (40.3)	143 (49)	1.42 (0.81 – 2.48)	
	**log-Additive**				
		62 (17.5)	292 (82.5)	0.67 (0.45 – 0.99)	**0.04295**
SNCA	**Codominant**				
**rs356181**	G/G	20 (32.3)	158 (54.1)	1	**0.002804**
	G/A	33 (53.2)	117 (40.1)	0.45 (0.25 – 0.82)	
	A/A	9 (14.5)	17 (5.8)	0.24 (0.09 – 0.61)	
	**Dominant**				
	G/G	20 (32.3)	158 (54.1)	1	**0.001615**
	G/A-A/A	42 (67.7)	134 (45.9)	0.4 (0.23 – 0.72)	
	**Recessive**				
	G/G-G/A	53 (85.5)	275 (94.2)	1	**0.02863**
	A/A	9 (14.5)	17 (5.8)	0.36 (0.15 – 0.86)	
	**Overdominant**				
	G/G-A/A	29 (46.8)	175 (59.9)	1	0.05826
	G/A	33 (53.2)	117 (40.1)	0.59 (0.34 – 1.02)	
	**log-Additive**				

		62 (17.5)	292 (82.5)	0.48 (0.31 − 0.73)	**0.000633**
SNCA	**Codominant**				
**rs8180209**	G/G	14 (22.6)	101 (34.6)	1	0.1218
	A/G	35 (56.5)	150 (51.4)	0.59 (0.30 − 1.16)	
	A/A	13 (21)	41 (14)	0.44 (0.19 − 1.01)	
	**Dominant**				
	G/G	14 (22.6)	101 (34.6)	1	0.05987
	A/G-A/A	48 (77.4)	191 (65.4)	0.55 (0.29 − 1.05)	
	**Recessive**				
	G/G-A/G	49 (79)	251 (86)	1	0.1833
	A/A	13 (21)	41 (14)	0.62 (0.31 − 1.23)	
	**Overdominant**				
	G/G-A/A	27 (43.5)	142 (48.6)	1	0.4662
	A/G	35 (56.5)	150 (51.4)	0.81 (0.47 − 1.42)	
	**log-Additive**				
		62 (17.5)	292 (82.5)	0.66 (0.44 − 0.99)	**0.04367**
SNCA	**Codominant**				
**rs3910105**	A/A	20 (32.3)	129 (44.2)	1	0.1314
	A/G	31 (50)	132 (45.2)	0.66 (0.36 − 1.22)	
	G/G	11 (17.7)	31 (10.6)	0.44 (0.19 − 1.01)	
	**Dominant**				
	A/A	20 (32.3)	129 (44.2)	1	0.08069
	A/G-G/G	42 (67.7)	163 (55.8)	0.6 (0.34 − 1.08)	
	**Recessive**				
	A/A-A/G	51 (82.3)	261 (89.4)	1	0.1326
	G/G	11 (17.7)	31 (10.6)	0.55 (0.26 − 1.17)	
	**Overdominant**				
	A/A-G/G	31 (50)	160 (54.8)	1	0.492
	A/G	31 (50)	132 (45.2)	0.82 (0.48 − 1.43)	
	**log-Additive**				
		62 (17.5)	292 (82.5)	0.66 (0.44 − 0.99)	**0.04393**
BIN3	**Codominant**				
**rs2280104**	C/C	46 (74.2)	172 (58.9)	1	0.05939
	T/C	15 (24.2)	107 (36.6)	1.91 (1.02 − 3.58)	
	T/T	1 (1.6)	13 (4.5)	3.48 (0.44 − 27.27)	
	**Dominant**				
	C/C	46 (74.2)	172 (58.9)	1	**0.02151**
	T/C-T/T	16 (25.8)	120 (41.1)	2.01 (1.08 − 3.71)	
	**Recessive**				
	C/C-T/C	61 (98.4)	279 (95.5)	1	0.2491
	T/T	1 (1.6)	13 (4.5)	2.84 (0.36 − 22.14)	
	**Overdominant**				
	C/C-T/T	47 (75.8)	185 (63.4)	1	0.05512
	T/C	15 (24.2)	107 (36.6)	1.81 (0.97 − 3.40)	
	**log-Additive**				
		62 (17.5)	292 (82.5)	1.9 (1.09 − 3.31)	**0.0175**
SH3GL2	**Codominant**				
**rs13294100**	T/T	25 (40.3)	75 (25.7)	1	0.0506
	T/G	22 (35.5)	146 (50)	2.21 (1.17 − 4.18)	
	G/G	15 (24.2)	71 (24.3)	1.58 (0.77 − 3.23)	
	**Dominant**				
	T/T	25 (40.3)	75 (25.7)	1	**0.0236**
	T/G-G/G	37 (59.7)	217 (74.3)	1.95 (1.10 − 3.46)	
	**Recessive**				
	T/T-T/G	47 (75.8)	221 (75.7)	1	0.9838
	G/G	15 (24.2)	71 (24.3)	1.01 (0.53 − 1.91)	
	**Overdominant**				
	T/T-G/G	40 (64.5)	146 (50)	1	**0.03623**
	T/G	22 (35.5)	146 (50)	1.82 (1.03 − 3.21)	
	**log-Additive**				
		62 (17.5)	292 (82.5)	1.33 (0.91 − 1.95)	0.1435
MIR4697	**Codominant**				
**rs329648**	C/C	33 (53.2)	129 (44.2)	1	0.05598
	T/C	27 (43.5)	128 (43.8)	1.21 (0.69 − 2.13)	
	T/T	2 (3.2)	35 (12)	4.48 (1.02 − 19.57)	

	**Dominant**				
	C/C	33 (53.2)	129 (44.2)	1	0.1948
	T/C-T/T	29 (46.8)	163 (55.8)	1.44 (0.83 − 2.49)	
	**Recessive**				
	C/C-T/C	60 (96.8)	257 (88)	1	**0.02114**
	T/T	2 (3.2)	35 (12)	4.09 (0.96 − 17.46)	
	**Overdominant**				
	C/C-T/T	35 (56.5)	164 (56.2)	1	0.967
	T/C	27 (43.5)	128 (43.8)	1.01 (0.58 − 1.76)	
	**log-Additive**				
		62 (17.5)	292 (82.5)	1.54 (0.99 − 2.41)	**0.04959**
SREBF1	**Codominant**				
**rs11868035**	A/A	44 (71)	224 (76.7)	1	0.09906
	G/A	14 (22.6)	64 (21.9)	0.9 (0.46 − 1.74)	
	G/G	4 (6.5)	4 (1.4)	0.2 (0.05 − 0.82)	
	**Dominant**				
	A/A	44 (71)	224 (76.7)	1	0.3457
	G/A-G/G	18 (29)	68 (23.3)	0.74 (0.40 − 1.37)	
	**Recessive**				
	A/A-G/A	58 (93.5)	288 (98.6)	1	**0.03342**
	G/G	4 (6.5)	4 (1.4)	0.2 (0.05 − 0.83)	
	**Overdominant**				
	A/A-G/G	48 (77.4)	228 (78.1)	1	0.9092
	G/A	14 (22.6)	64 (21.9)	0.96 (0.50 − 1.86)	
	**log-Additive**				
		62 (17.5)	292 (82.5)	0.66 (0.40 − 1.11)	0.1264

Table S5 Clinical characteristics of patients with iRBD stratified by *SH3GL2* rs13294100 and *COMT* rs165599 genotypes

iRBD	***SH3GL2* rs13294100**		***P* value**	***COMT* rs165599**		***P* value**
	**TT (n = 21)**	**TG+GG (n = 31)**		**GG (n = 6)**	**GA+AA (n = 46)**	
**Age (y)**	62.84±8.58	63.29±8.95	0.855	66.95±6.85	62.61±8.88	0.256
**Gender, n (%)**						
Male	14 (66.67%)	19 (61.29%)	0.693	6 (100.00%)	27 (58.70%)	0.127
Female	7 (33.33%)	12 (38.71%)		0 (0.00%)	19 (41.30%)	
**MDS-UPDRS II**	0.89±1.70	1.72±3.26	0.322	0.4±0.55	1.49±2.85	0.404
**RBDSQ**	8.30±2.15	8.68±2.42	0.572	10.67±1.21	8.21±2.26	**0.013**
**MMSE**	28.35±1.66	28.30±2.37	0.931	28.33±1.51	28.32±2.16	0.986
**MoCA**	26.45±2.44	25.59±3.30	0.332	27.17±1.72	25.78±3.08	0.289
**HAMA**	5.22±3.89	6.52±4.22	0.313	6.83±3.54	5.80±4.19	0.573
**HAMD**	2.42±2.43	5.33±5.57	**0.021**	5.67±3.27	3.90±4.91	0.400
**SS-16**	7.65±2.92	7.73±3.55	0.935	6.50±3.08	7.88±3.28	0.341
**SCOPA-AUT**	7.10±4.61	7.46±5.33	0.806	10.00±7.16	6.93±4.60	0.161
**NMSQ**	6.32±2.96	7.93±3.91	0.134	9.67±4.13	6.93±3.45	0.082

iRBD, idiopathic REM sleep behavior disorder; LEDD, levodopa equivalent daily dose; H-Y, Hoehn-Yahr; MDS-UPDRS, Movement Disorder Society-sponsored revision of the Unified Parkinson's Disease Rating Scale; RBDSQ, REM Sleep Behavior Disorder Screening Questionnaire; MMSE, Mini-Mental State Examination; MoCA, Montreal Cognitive Assessment; HAMA, Hamilton Anxiety Scale; HAMD, Hamilton Depression Scale; SS-16, Sniffin' Sticks 16-item odor identification test; SCOPA-AUT, Scale for Outcomes in PD-Autonomic; NMSQ, Non-Motor Symptom Questionnaire.

The bold emphasis in the table indicates $P < 0.05$.

Figure S6 Sleep parameters in iRBD patients compared across *SH3GL2* rs13294100 and *COMT* rs165599 genotypes

iRBD	*SH3GL2* rs13294100		*P* value	*COMT* rs165599		*P* value
	TT (n = 24)	TG+GG (n = 28)		GG (n = 8)	GA+AA (n = 44)	
**Total sleep time (min)**	375.02±82.76	320.80±86.38	**0.026**	297.13±101.20	354.68±83.93	0.090
**Sleep latency (min)**	43.73±48.99	50.23±49.79	0.638	67.56±58.91	43.53±46.88	0.206
**Microarousal index**	20.23±16.31	16.66±9.59	0.333	18.48±11.23	18.28±13.54	0.970
**Sleep Efficiency (%)**	60.17±14.46	56.58±16.3	0.409	47.33±18.38	60.22±14.19	**0.028**
**Wake (min)**	138.79±93.78	122.23±57.3	0.439	179.75±86.96	120.80±71.20	**0.042**
**REM (%)**	17.15±6.34	17.78±9.61	0.778	15.89±9.80	17.78±7.96	0.553
**Stage 1(%)**	5.26±3.84	7.08±7.24	0.255	5.20±3.27	6.43±6.31	0.596
**Stage 2(%)**	61.55±10.59	60.84±12.01	0.821	63.18±12.73	60.80±11.11	0.589
**Stage 3(%)**	16.05±8.33	14.30±6.86	0.408	15.74±9.65	14.99±7.24	0.800
**AHI**	2.64±5.18	2.18±5.69	0.764	3.64±6.89	2.17±5.17	0.486
**Oxygen desaturation events**	83.67±92.61	91.04±99.30	0.784	138.38±124.99	78.41±87.67	0.102
**Average heart rate**	65.96±10.66	64.39±6.45	0.521	63.00±5.74	65.43±8.92	0.490
**PLM index**	9.53±13.48	16.34±26.13	0.255	14.78±19.22	12.91±21.89	0.823

iRBD, idiopathic REM sleep behavior disorder; AHI, apnea-hypopnea index; PLM: periodic limb movement. The bold emphasis in the table indicates $P < 0.05$.

Table S7 Clinical manifestations of different genotypes of PD

PD	*SH3GL2* rs13294100		*P* value	*COMT* rs165599		*P* value
	**TT (n = 71)**	**TG+GG (n = 198)**		**GG (n = 62)**	**GA+AA (n = 207)**	
**Age (y)**	63.36±10.07	63.24±8.40	0.930	62.01±8.31	63.65±8.99	0.202
**Gender, n (%)**						
Male	37 (52.11%)	104 (52.53%)	0.952	32 (51.61%)	109 (52.66%)	0.885
Female	34 (47.89%)	94 (47.47%)		30 (48.39%)	98 (47.34%)	
**LEDD**	374.97±245.57	383.51±280.82	0.836	326.10±281.61	396.94±267.10	0.107
**H-Y stage**	1.82±0.86	1.73±0.87	0.458	1.61±0.92	1.79±0.85	0.148
**MDS-UPDRS II**	12.35±7.05	11.11±6.27	0.186	10.94±5.93	11.58±6.66	0.515
**MDS-UPDRS III**	30.51±17.43	27.35±15.07	0.167	28.68±15.17	28.02±15.95	0.782
**RBDSQ**	4.94±4.09	4.40±3.24	0.349	4.20±3.56	4.64±3.45	0.403
**MMSE**	26.71±3.20	27.32±2.88	0.157	26.89±2.64	27.23±3.06	0.455
**MoCA**	22.62±4.86	23.11±4.27	0.448	22.46±4.12	23.14±4.52	0.328
**HAMA**	7.02±7.05	8.26±7.42	0.289	9.30±8.52	7.52±6.93	0.158
**HAMD**	5.13±4.83	4.98±5.50	0.847	5.87±4.97	4.77±5.40	0.187
**SS-16**	7.21±3.30	7.17±3.15	0.932	7.40±3.13	7.12±3.20	0.580
**SCOPA-AUT**	11.31±7.81	10.92±7.71	0.727	8.95±6.56	11.65±7.95	**0.020**
**NMSQ**	8.28±4.51	7.99±4.16	0.636	8.05±4.23	8.07±4.26	0.980

PD, Parkinson's disease; LEDD, levodopa equivalent daily dose; H-Y, Hoehn-Yahr; MDS-UPDRS, Movement Disorder Society-sponsored revision of the Unified Parkinson's Disease Rating Scale; RBDSQ, REM Sleep Behavior Disorder Screening Questionnaire; MMSE, Mini-Mental State Examination; MoCA, Montreal Cognitive Assessment; HAMA, Hamilton Anxiety Scale; HAMD, Hamilton Depression Scale; SS-16, Sniffin' Sticks 16-item odor identification test; SCOPA-AUT, Scale for Outcomes in PD-Autonomic; NMSQ, Non-Motor Symptom Questionnaire.

The bold emphasis in the table indicates $P < 0.05$.

Figure S8 Sleep structure in different genotypes of PD

PD	*SH3GL2* rs13294100		*P* value	*COMT* rs165599		*P* value
	TT (n = 24)	TG+GG (n = 56)		GG (n = 13)	GA+AA (n = 67)	
**Total sleep time (min)**	321.15 ± 87.16	317.02 ± 119.42	0.879	276.92 ± 112.48	326.28 ± 108.78	0.140
**Sleep latency (min)**	66.40 ± 80.90	55.38 ± 68.14	0.533	55.08 ± 57.82	59.38 ± 74.63	0.845
**Microarousal index**	13.52 ± 9.13	15.29 ± 7.86	0.380	15.02 ± 7.73	14.71 ± 8.39	0.904
**Sleep Efficiency (%)**	54.53 ± 15.01	52.39 ± 19.66	0.598	47.41 ± 21.47	54.12 ± 17.63	0.229
**Wake (min)**	132.42 ± 84.58	141.58 ± 84.44	0.659	165.35 ± 96.98	133.57 ± 81.06	0.215
**REM (%)**	14.55 ± 9.11	17.15 ± 8.60	0.228	17.92 ± 8.03	16.06 ± 8.95	0.489
**Stage 1(%)**	4.76 ± 4.44	6.41 ± 5.69	0.212	3.48 ± 4.41	6.39 ± 5.43	0.074
**Stage 2(%)**	62.84 ± 14.20	60.67 ± 13.66	0.524	63.31 ± 11.51	60.94 ± 14.22	0.574
**Stage 3(%)**	17.85 ± 9.89	15.87 ± 11.66	0.470	15.30 ± 8.29	16.70 ± 11.64	0.681
**AHI**	3.48 ± 6.82	4.87 ± 9.16	0.507	4.43 ± 8.07	4.46 ± 8.65	0.991
**Oxygen desaturation events**	72.88 ± 86.94	76.54 ± 79.30	0.855	64.85 ± 58.40	77.49 ± 85.04	0.610
**Average heart rate**	64.65 ± 8.76	64.62 ± 6.76	0.985	63.62 ± 7.32	64.83 ± 7.39	0.589
**PLM index**	13.09 ± 29.98	17.10 ± 35.13	0.627	19.27 ± 33.66	15.24 ± 33.72	0.695

PD, Parkinson's disease; AHI, apnea-hypopnea index; PLM: periodic limb movement.
